# Pitch and Plasticity: Insights from the Pitch Matching of Chords by Musicians with Absolute and Relative Pitch

**DOI:** 10.3390/brainsci3041615

**Published:** 2013-12-03

**Authors:** Neil M. McLachlan, David J. T. Marco, Sarah J. Wilson

**Affiliations:** Melbourne School of Psychological Sciences, The University of Melbourne, Victoria 3010, Australia; E-Mails: dmarco@unimelb.edu.au (D.J.T.M.); sarahw@unimelb.edu.au (S.J.W.)

**Keywords:** pitch, absolute pitch, concurrent pitch, neurocognitive model, recognition

## Abstract

Absolute pitch (AP) is a form of sound recognition in which musical note names are associated with discrete musical pitch categories. The accuracy of pitch matching by non-AP musicians for chords has recently been shown to depend on stimulus familiarity, pointing to a role of spectral recognition mechanisms in the early stages of pitch processing. Here we show that pitch matching accuracy by AP musicians was also dependent on their familiarity with the chord stimulus. This suggests that the pitch matching abilities of both AP and non-AP musicians for concurrently presented pitches are dependent on initial recognition of the chord. The dual mechanism model of pitch perception previously proposed by the authors suggests that spectral processing associated with sound recognition primes waveform processing to extract stimulus periodicity and refine pitch perception. The findings presented in this paper are consistent with the dual mechanism model of pitch, and in the case of AP musicians, the formation of nominal pitch categories based on both spectral and periodicity information.

## 1. Introduction

A small percentage of people possess absolute pitch (AP), or the ability to identify single pitches using musical note names without a reference pitch [1]. Since a neurocognitive account of AP should be informed by models of pitch processing more generally, we will briefly review this literature before introducing a model of AP that emerges from recent work investigating the role of recognition mechanisms in pitch perception [2,3,4,5,6].

### 1.1. The Evolution of Pitch Models

In early pitch models, pitch height was believed to correspond to the region of maximum excitation on the basilar membrane of the cochlea [7]. However, these models could not explain the perception of a virtual pitch at the fundamental frequency of harmonic complexes when this tone component was absent from the stimulus. Thus, in the mid to late 20th century, harmonic template matching models and temporal waveform processing models of pitch were developed to explain virtual pitch perception [7].

In harmonic template models [8,9] a number of tones tuned to a subset of the harmonic series would be sufficient to activate a harmonic template and generate pitch salience at the fundamental frequency proportional to the salience of the harmonics [10]. Temporal processing models were initially based on autocorrelation functions that identify the period of the waveform [11], and could explain virtual pitch perception as a sub-harmonic response in autocorrelation functions when summed over the outputs of multiple auditory filter channels [12]. A strong argument in favor of autocorrelation models was that they could produce much finer pitch resolution than template matching models. Computation of inter-spike time delays on the auditory nerve produces autocorrelation like behaviors that were initially thought to be able to explain pitch perception [13], although later periodicity models were based on tightly tuned neural responses to periodicity in the inferior colliculus [14,15].

Moore [16] suggested that both spectral and temporal mechanisms are involved in pitch processing, with periodicity providing higher pitch resolution at lower frequencies for which auditory nerve responses are phase locked to the stimulus waveform. Later, Oxenham *et al.* [17] reported robust pitch perception at frequencies well above the limit of phase locking on the auditory nerve, and postulated that low familiarity with high frequency stimuli may have contributed to poorer pitch accuracy for stimuli at high frequencies in earlier studies. Other researchers suggested that either spectral or temporal mechanism may be used, depending on whether the stimuli are resolved by the peripheral auditory system [18], although it was later shown that spectral and temporal pitch mechanisms cannot operate independently [19]. Furthermore, Smith *et al.* [20] showed that either periodicity or spectral information can dominate in the perception of ambiguous pitch stimuli, with periodicity becoming more important when spectral cues are degraded below the resolution of a critical band. Notwithstanding the proposition that both spectral and temporal processing mechanisms are necessary for fine pitch processing has generally been considered to lack parsimony [7].

### 1.2. Concurrent Pitch Processing

The accuracy of pitch height judgments is greatly degraded by the presence of a concurrent pitch [21]. Harmonic template matching and periodicity models of pitch face substantial challenges in predicting pitch matching and segregation behavior for concurrent pitches. Harmonic template matching models predict that strong virtual pitch percepts should emerge at the frequency of the lowest common factor of the harmonics in chords [22]. However, musicians do not mistake intervals such as a minor 3rd (frequency ratio of approximately 6/5) with a single pitch at 1/5 the frequency of the lowest frequency tone component in the chord. Pitch matching data for chords shows that accuracy is better rather than worse for chords with tunings close to simple frequency ratios [5,23].

Micheyl *et al.* [24] showed that good pitch discrimination of harmonic complexes in the presence of masking tones could only be achieved when several harmonics belonging to the target pitch were likely to be salient in tonotopic spike rate representations on the auditory nerve, providing further support for the importance of spectral information in concurrent pitch processing. Furthermore, it is unlikely that periodicity information is used to segregate concurrent vowels presented at different pitches [25,26,27], because this information takes longer to accumulate than the duration of many speech phonemes [28,29].

More recently it has been shown that the identification of concurrent vowels presented at different pitches improves with training on the stimulus set [30]. Consistent with this, pitch matching accuracy for concurrent pitches is better for more common chords, and improves with music training [5]. These findings, together with computational modeling by McLachlan [4] suggest that long-term memory templates of spectral information associated with pitch intervals may be used to segregate and group neural activity across auditory filter channels prior to fine pitch processing by temporal waveform information. The finding that musicians are more accurate at matching the highest pitch component of chords [5] further suggests that neural activity is first integrated for the highest pitch, and then attention is sequentially shifted to integrate information for the other pitches. This is consistent with the practice of placing melodies at the highest pitch of accompanying chords [31], and the finding that brain stem electrical activity is more strongly phase locked to the harmonics of the higher pitch in chords in musicians [32].

### 1.3. Models of Absolute Pitch

In musicians, AP ability has been explained using a hierarchical, two-component model, where absolute representation of pitch in long-term memory is the first component, and pitch labeling is the second component [33]. The absolute representation of pitch in fixed categories is considered common to all humans and some other species [34,35,36,37,38]. In contrast, the associations between pitch representations and labels that allow tones to be organized into nominal categories are thought to be acquired during a critical or sensitive period of development, and are only evident in select individuals [37].

Behavioral and neurophysiological findings have identified quasi-absolute pitch (QAP) musicians as having a restricted AP ability, typically for more commonly heard pitches such as the white notes of the piano or the strings of their instrument [39,40]. QAP musicians often rely on relative pitch (RP) judgments for pitches that they cannot automatically label [40], and so have fewer associations between stimulus representations and verbal labels for pitch categories. Increased timbral dependency in QAP musicians may also reflect a restricted range of absolute representations (or templates) that is closely aligned with the timbre of the instrument on which they trained. Therefore QAP musicians may have fewer templates to associate with verbal labels than AP musicians. Consistent with relationships between AP ability and specific instrumental timbres, Wilson *et al.* [41] showed that ongoing training with a “fixed pitch” instrument such as the keyboard was an important factor in developing and maintaining AP skill, reflecting the need to maintain long-term memory templates through training.

The proposition that pitch processing is initiated by spectral recognition mechanisms would enable AP and QAP musicians to rapidly associate verbal labels of pitch to spectral representations of the stimuli, just as people more generally associate verbal labels to familiar sounds. In support of this Hsieh and Saberi [42] observed that AP participants have the same pitch just noticeable difference limens as RP participants, however the AP participants reached these discrimination limens after only four waveform cycles for western musical pitches. In contrast, pitch discrimination limens for RP musicians improve as stimulus duration increases over the first ten waveform cycles, likely due to the sharpening of pitch representations as more temporal waveform information becomes available [2,16,42].

### 1.4. The Present Research

To investigate whether categorical processing of pitch by AP and QAP musicians commences with sound recognition mechanisms that are common to all musicians, we compared data for RP musicians from an earlier pitch matching study of music chords [5], with new data from AP and QAP musicians for the same chords. In contrast to the idea that AP musicians can perceive pitches independently of their musical or timbral context, we predicted that pitch matching accuracy would be better across all three musician groups for more commonly used, familiar chords, and better for the highest target pitch across all chords. We also predicted that AP musicians would better match pitches in more spectrally dense stimuli (e.g., 3-pitch as compared to 2-pitch chords) than other musicians, suggesting that they have finer resolution spectral representations that would enable them to rapidly identify and label individual pitches based on spectral information alone.

We asked highly trained musicians with varying AP skill to match all the pitches of a series of 2- and 3-pitch western music chords using a variable pitch sine tone. The chords differed in their usage in common western music [43] and participants were asked to rate the familiarity of each stimulus. We first hypothesized that the pitch matching accuracy of all musicians would be strongly correlated with their familiarity ratings for the chords (Hypothesis 1a), and that pitch matching accuracy for high familiarity chords would be better than for low familiarity chords (Hypothesis 1b). Second, consistent with our previous finding for RP musicians, we hypothesized that pitch matching accuracy for the highest pitch in each chord would be better than all other pitches for AP and QAP musicians as well (Hypothesis 2). Third, we hypothesized that for spectrally dense stimuli, the pitch matching accuracy of AP musicians would be better than QAP and RP musicians (Hypothesis 3).

## 2. Experimental Section

### 2.1. Participants

Thirty-three adults [24 females; mean age 25 year (SD = 12.8)] took part in this study. Participants comprised undergraduate and postgraduate students from The University of Melbourne (Victoria, Australia) and The Melbourne Conservatorium of Music (Victoria, Australia), as well as adults recruited from the community who were known to have AP by the researchers. The mean number of year of formal education was 19.2 year (SD = 7.7). The study was conducted in accordance with ethical guidelines established by The University of Melbourne. Information about the study was provided to participants and written informed consent was obtained.

Participants were tested for the presence of AP using an established pitch naming task [40,41]. This task comprised a digital audio recording of 50 pseudo-randomly selected piano tones played sequentially, each 500 ms in duration played 2.5 s apart. Participants were asked to identify the pitch chroma of each tone they heard to the best of their ability, and write it down on a piece of paper. Participants were given a score out of 50 and were not permitted semitone errors to better discriminate between AP and QAP musicians. All RP musicians achieved pitch naming scores that were at the level of chance performance. A *k*-means cluster analysis was conducted on the remaining pitch naming scores to identify 2 non-overlapping QAP and AP groups (Table 1). Since AP musicians usually have high levels of music training, only RP participants who reported receiving more than 15 year of formal training on pitched instruments or voice in a western music tradition were included in this study. All participants reported having normal hearing, and no serious neurological conditions.

**Table 1:** The musical skill and training of the musician groups. RP, relative pitch; QAP, quasi-absolute pitch; AP, absolute pitch.

Musician Group	*N*	Pitch Naming Performance/50	Mean Year of Music Training (SD)
RP	12	0–7	19.04 (4.9)
QAP	12	10–35	18.0 (9.7)
AP	9	40–50	21.2 (8.2)

### 2.2. Materials

The stimuli from McLachlan *et al.* [5] were used in this study. They comprised (1) pure tones; (2) odd harmonic complexes comprising a fundamental plus equal-amplitude harmonics at three and five times the frequency of the fundamental; and (3) full harmonic complexes comprising a fundamental plus five equal-amplitude harmonics. Six 2-pitch and six 3-pitch chords with intervals of up to eight semitones were created from each stimulus type (Table 2), generating 36 chords in all. These chords provide a representative sample of common and uncommon chords in accordance with chord usage in Western music [43]. Each chord was presented sufficient times to allow pitch matching of each pitch in the chord in separate presentations in different blocks (see procedure). In addition to the chords, three single pitch stimuli of each stimulus type were presented for comparison with pitch matching distributions for the chords (nine 1-pitch stimuli in all). This produced a total of 99 stimulus presentations. The pitches of the stimuli were evenly distributed within the Western chromatic scale over the range of 220–466 Hz (A3 to A#4).

### 2.3. Procedure

For each stimulus presentation, participants were first asked to match one of the pitches in the stimulus, and then rate the familiarity and dissonance of the stimulus on separate 5-point Likert-type scales. All stimuli were presented to participants individually in an anechoic chamber at 70 ± 2 dB sound pressure level through two loudspeakers located on either side of a computer monitor (1 m in front and 0.5 m apart). Trials were presented randomly over three blocks of 25 trials and one block of 24 trials, with each block lasting approximately 10 min. Breaks were provided between blocks to minimise fatigue effects.

**Table 2:** Intervals used in this study showing their frequency differences and common music names.

Interval (Semitones)	Frequency Difference (%)		Chord Names
2	12.2		major 2nd
3	18.9		minor 3rd
4	26		major 3rd
6	41.4		tritone
7	49.8		perfect 5th
8	58.7		minor 6th
2 and 7	12.2	49.8	suspended 2nd triad
3 and 6	18.9	41.4	diminished 5th triad
3 and 7	18.9	49.8	minor triad
4 and 6	26	41.4	flattened 5th triad
4 and 7	26	49.8	major triad
4 and 8	26	58.7	augmented 5th triad

**Figure 1:**
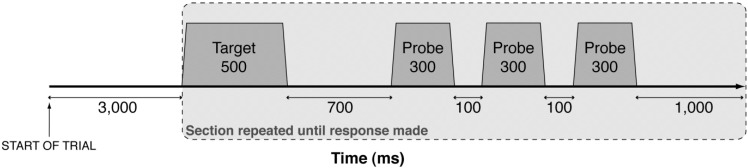
A schematic representation of the presentation of auditory stimuli. Each target stimulus and probe were synthesised with 30 ms linear onset and offset ramps and presented in a continuous sequence (*shaded box*) until participants matched the pitch of the probe to the target. Probes were synthesised in real-time at frequencies governed by participant movement of the computer mouse. Axis not shown to scale.

The pitch matching task was adapted from Moore and Glasberg [44]. Before the presentation of each chord, participants were informed of the number of pitches (one, two or three) and the target pitch they were required to match (lowest, middle or highest). Target stimuli were followed by a set of three pure tone probes, as shown in Figure 1. Bidirectional lateral movement of a computer mouse by the participant instantly altered the pitch of the probe tones (right movement increased pitch). The target stimulus and probe tones were repeated until participants clicked the mouse to indicate when they thought the probe tone matched the target, and the cycle was terminated. Purpose-built computer software was used to present the stimuli and record task responses. The software distributed 800 screen pixels evenly between 200 and 500 Hz, providing a frequency resolution of 0.375 Hz per pixel. Pitch matching of all component pitches in each chord was pseudo-randomly ordered so that consecutive presentations of the same chord were avoided.

At the completion of each pitch matching trial a rating screen appeared on the computer monitor to grade the dissonance and familiarity of the target stimuli. Two separate 5-point Likert-type scales measured perceived familiarity (1 = not familiar, 5 = very familiar) and perceived dissonance (1 = not dissonant, 5 = very dissonant). Dissonance data are not included in the present analyses.

Prior to the experimental trials, participants completed a questionnaire to collect demographic, health, and musical background data [45]. They then completed three practice trials on 2- and 3- pitch chords with feedback to ensure adequate task comprehension, followed by a series of screening trials using pure tone stimuli. The screening continued up to a maximum of 10 trials with feedback until participants had accurately pitch matched a pure tone to within two semitones on three successive trials. All participants successfully completed the screening task.

### 2.4. Pitch Matching Accuracy and Data Analysis

There are a number of potential sources of error in the pitch matching of chords that can cause large skews in distributions and have complex influences on statistical measures of centroid such as means [5]. These include the tendency to match at frequencies between stimulus pitches [5,21], and matches at the wrong pitch in the stimulus. To avoid the influence of these skews, the percentage of pitch matching responses within a tolerance around the target pitch was measured (the percentage of correct pitch matches). To facilitate comparison of results with a previous study by the authors [5], the tolerance was based on the pitch matching performance of highly trained RP musicians for single pitch stimuli. A tolerance of ±0.41 semitones was two standard deviations of the mean of the pitch matching distribution for these musicians. The AP and QAP musician pitch matching distributions for single pitch stimuli were close to the RP distribution (two standard deviations of ±0.48 and ±0.72 semitones respectively). In keeping with data reported by Hsieh and Saberi [42], a Levine’s test revealed no significant differences in the variances of these distributions [*F*(2, 291) = 2.747, *p* > 0.05].

We then tested whether stimulus type (pure tones, odd harmonic, and full harmonic complexes) affected familiarity or pitch matching accuracy using two mixed between and within groups analyses of variance (ANOVA) with independent variables of musician group (three levels) and stimulus type (three levels). No interactions were found for familiarity [*F*(3.5, 52.2) = 0.71, *p* = 0.57] or pitch matching accuracy [*F*(4, 60) = 1.05, *p* = 0.39], and no main effect for stimulus type was found for familiarity [*F*(1.7, 52.2) = 1.55, *p* = 0.22] or pitch matching accuracy [*F*(2, 60) = 3.08, *p* = 0.05], so the data were collapsed across stimulus type in all subsequent analyses.

To address Hypothesis 1a, that pitch matching accuracy would be correlated with chord familiarity ratings, the percentage of correct pitch matches for each chord was computed and correlated with the mean familiarity ratings of chords for each participant group using Pearson coefficients. To address hypotheses 1b, that pitch matching accuracy for high familiarity chords would be better than for low familiarity chords, the chords were first divided into similarly sized groups of high and low familiarity. This grouping was confirmed for the participants in this study by comparison of the mean familiarity ratings for each chord with the median familiarity rating score of 4 across all participants by single sample *t*-tests. Table 3 lists the chords in descending order of the t statistic, with the chords in the top half of Table 3 belonging to the high familiarity chord group. Pitch matching accuracy was then compared using an ANOVA with the between groups variables of chord familiarity (high and low) and musician group (RP, QAP and AP).

**Table 3:** Comparison of the mean familiarity ratings for each chord with the median rating score (4) across all participants by single sample *t*-tests. Items are listed in descending order of the *t* statistic, with values in bold indicating chords included in the high familiarity chord group.

Chord	Semitone Intervals	Familiarity Rating Mean (SD)	*t*	Effect size (*R*^2^)
major triad	4 and 7	4.45 (0.79)	9.89	0.25
major 3rd	4	4.32 (0.93)	4.87	0.11
perfect 5th	7	4.21 (0.94)	3.11	0.05
minor 6th	8	4.13 (1.01)	1.83	0.02
minor triad	3 and 7	4.02 (1.07)	0.38	0.00
minor 3rd	3	3.85 (1.05)	−1.97	0.02
tritone	6	3.61 (1.17)	−4.64	0.10
diminished 5th triad	3 and 6	3.64 (1.11)	−5.55	0.09
suspended 2nd triad	2 and 7	3.60 (1.21)	−5.66	0.10
augmented 5th triad	4 and 8	3.48 (1.30)	−6.92	0.14
major 2nd	2	3.31 (1.39)	−6.96	0.20
flattened 5th triad	4 and 6	3.35 (1.28)	−8.74	0.21

Due to the different number of pitches in 2-, and 3-pitch chords, two separate mixed between and within groups ANOVAs were used to address Hypothesis 2, that pitch matching accuracy for the highest pitch in each chord would be better than all other pitches across all musician groups. The within group variable was target pitch position (high and low for 2-pitch chords, and high, middle and low for 3-pitch chords), and the between groups variable was musician group (RP, QAP and AP). This analysis was also used to address Hypothesis 3, that for more spectrally dense 3-pitch chords the pitch matching accuracy of AP musicians would be better than QAP musicians, who in turn would be better than RP musicians.

## 3. Results and Discussion

**Figure 2:**
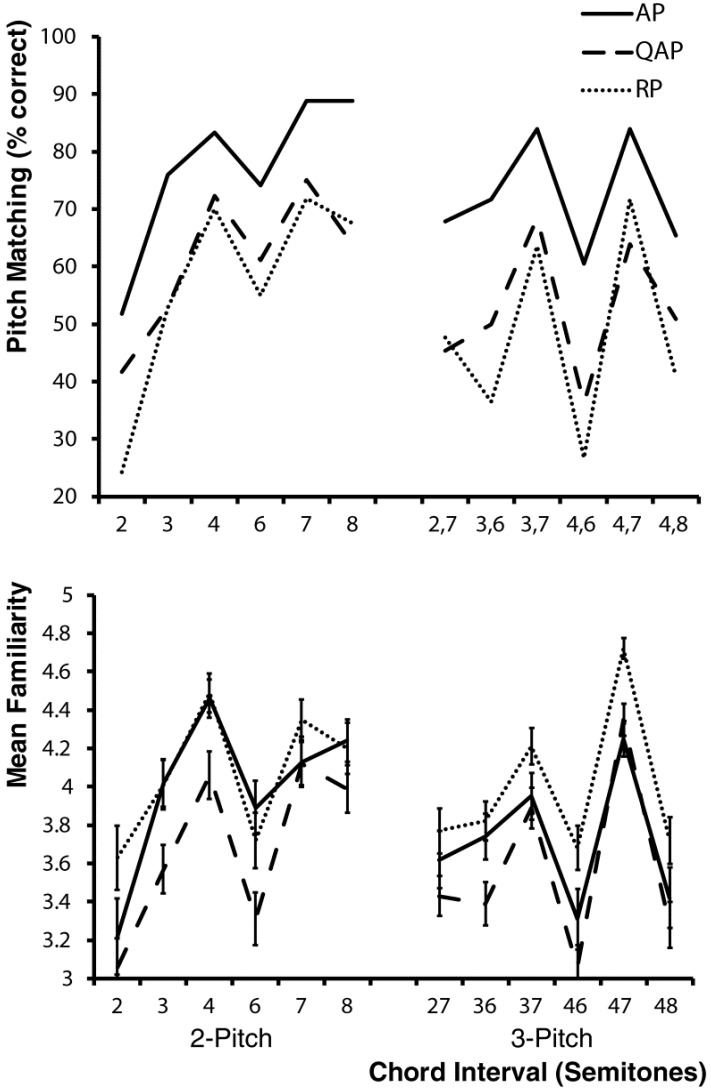
Pitch matching accuracy and mean familiarity ratings of the three musician groups for the 2- and 3-pitch chords described in Table 2.

Figure 2 shows that the pitch matching accuracy of all three musician groups was strongly related to their mean familiarity ratings for the chords, and in confirmation of Hypothesis 1a Pearson correlations of mean familiarity and pitch matching accuracy were all similarly strong, RP (*R* = 0.87, *p* < 0.01), QAP (*R* = 0.86, *p* < 0.01) and AP (*R* = 0.93, *p* < 0.01). In support of Hypothesis 1b, the mixed between and within groups ANOVA revealed a significant main effect for chord familiarity, [*F*(1, 30) = 150.23, *p* < 0.001, partial η^2^ = 0.83] in which the mean pitch matching accuracy for high familiarity chords (mean = 71.2, SD = 24.9) was greater than for low familiarity chords (mean = 48.9, SD = 24.7), but there was no main effect for musician group or interaction between chord familiarity and musician group (both *p* > 0.05).

**Figure 3:**
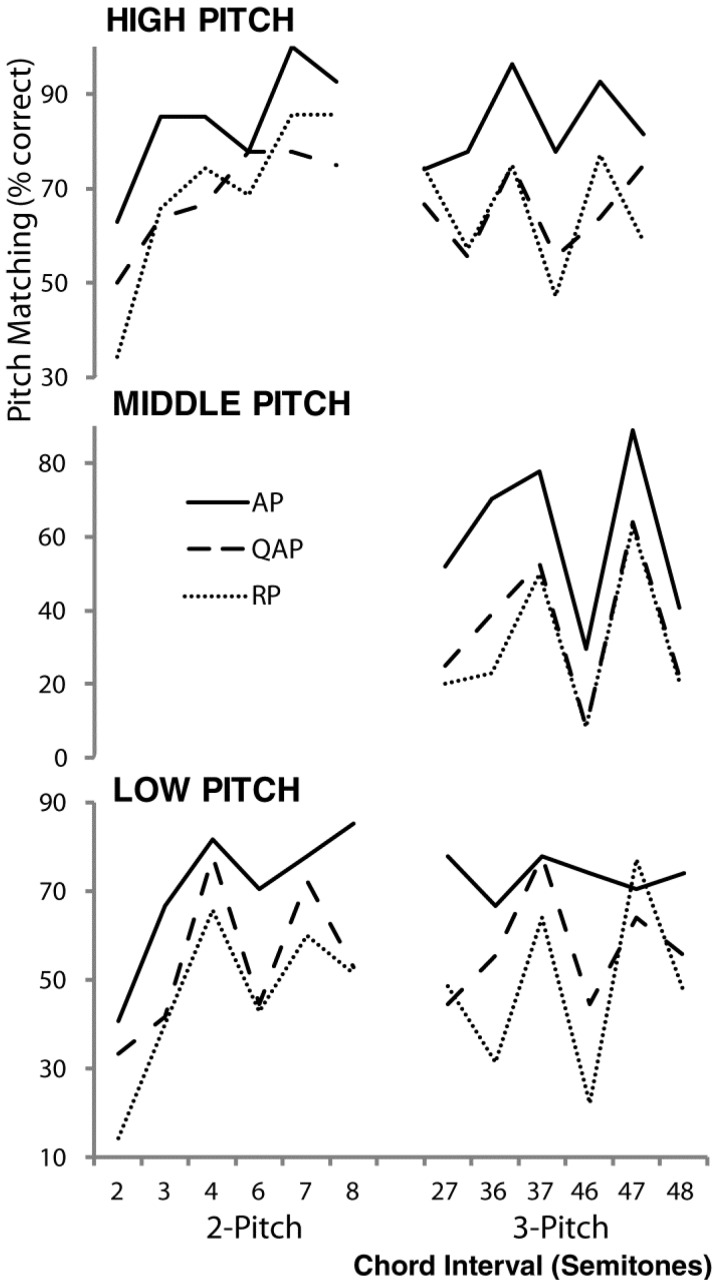
Pitch matching accuracy of the three musician groups for the high, middle and low pitch of the 2- and 3-pitch chords described in Table 2.

Figure 3 shows pitch matching accuracy for each target pitch and musician group. Overall the pitch matching accuracy for the high target pitch was greater than the other pitches for each musician group. Hypothesis 2, that pitch matching accuracy for the highest pitch in each chord would be better than for other pitches, was supported by main effects for target pitch position in the ANOVAs for both 2- and 3-pitch chords, [*F*(1, 30) = 23.58, *p* < 0.001, partial η^2^ = 0.44 and *F*(2, 60) = 47.80, *p* < 0.001, partial η^2^ = 0.61 respectively]. In both instances pitch matching accuracy was significantly better for the highest pitch target. For the 3-pitch chords, planned repeated contrasts revealed that pitch matching performance was worse for the middle target pitch when compared to both the high target pitch [*F*(1, 30) = 20.41, *p* < 0.001, partial η^2^ = 0.41] and the low target pitch [*F*(1, 30) = 27.52, *p* < 0.001, partial η^2^ = 0.49].

Finally, in partial support of Hypothesis 3 that for spectrally dense 3-pitch chords, the pitch matching accuracy of AP musicians would be better than QAP musicians, who in turn would be better than RP musicians, the ANOVA showed a trend toward significance for musician group for 3-pitch chords [*F*(2, 30) = 2.88, *p* = 0.07, partial η^2^ = 0.16], but not 2-pitch chords [*F*(2, 30) = 2.19, *p* = 0.13, partial η^2^ = 0.13]. Simple planned contrasts showed significantly better pitch matching performance by AP musicians when compared to RP musicians (*p* < 0.05), and a trend when compared to QAP musicians (*p* = 0.07) for the 3-pitch chords. No interactions were found between target position and training groups for both 2- and 3-pitch chords (both *p* > 0.05).

**Figure 4:**
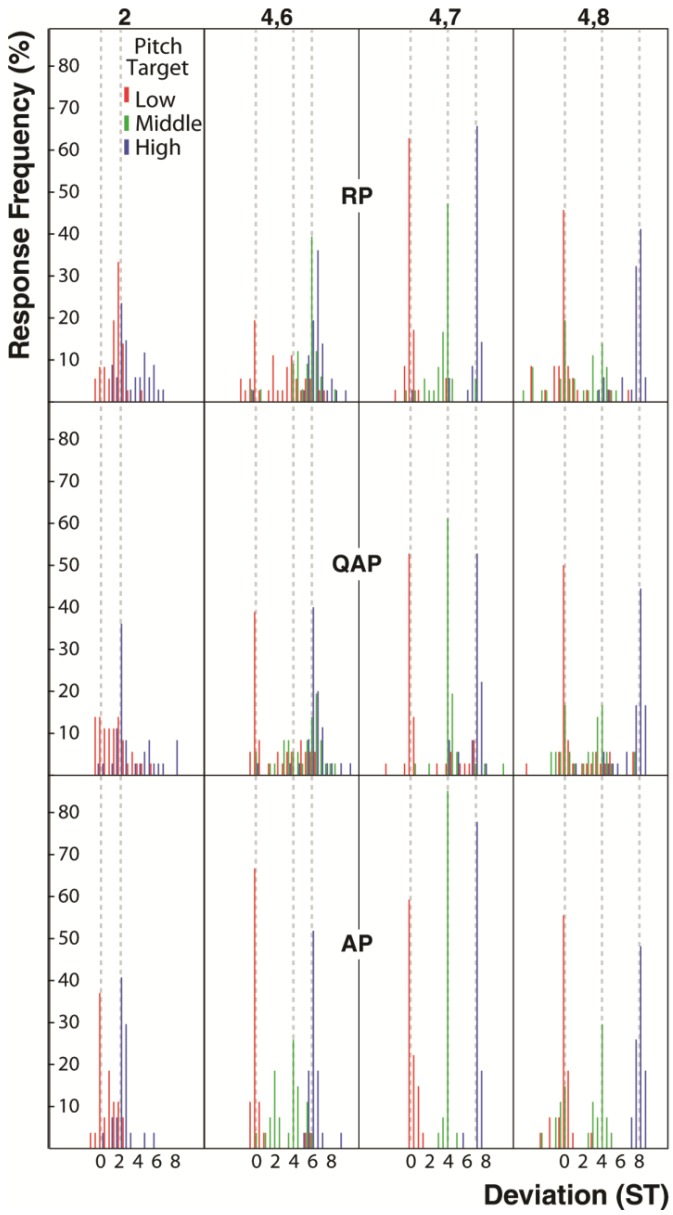
Histograms of pitch matching performance by each musician group for the three chords rated as most unfamiliar by all musicians (2nd, 2 semitones; flattened 5th triad, 4 and 6 semitones; augmented 5th triad, 4 and 8 semitones) contrasted with the chord rated as most familiar (major triad, 4 and 7 semitones). Grey dashed lines demark the frequency of target pitches.

The lack of difference in the variances of pitch matching distributions between musician groups for single pitches [*F*(2, 30) = 0.29, *p* = 0.75] is consistent with previous research that shows that AP musicians do not have better pitch discrimination than RP musicians for single pitches [46]. The trend for better pitch matching performance by the AP musicians for 3-pitch chords but not 2-pitch chords or single pitches, despite no differences in the levels of music training (Table 1) or chord familiarity ratings [3-pitch, *F*(2, 30) = 0.72, *p* = 0.72, partial η^2^ = 0.05] suggests that AP musicians may possess chord templates with more finely tuned spectral information than the other musician groups that assist them in disambiguating complex spectral information. To explore this idea further we examined the pitch matching responses for the mostly closely spaced 2-pitch chord, the major second (2-semitones), shown in the histograms for each musician group in Figure 4. Consistent with Hypothesis 3, only the AP musicians showed two distinct pitch matching distributions for the higher and lower target pitches of this chord. Post hoc analysis showed that the pitch matching accuracy for the highest and lowest pitches in the chord were significantly different for the RP musicians [*t*(11) = 2.283, *p* < 0.05], but not the QAP and AP musicians. In other words, the AP musicians performed equally well for both target positions, indicating that they were able to resolve the two pitches, as suggested by inspection of Figure 4. Note that the QAP pitch matching accuracy for the two semitone chord was intermediate between AP and RP accuracy (Figure 3), but the difference between the distributions for the two target pitches did not reach significance.

The proposition that AP musicians possess chord templates with more finally tuned spectral information (Hypothesis 3), suggests that compared to other musicians their pitch matching accuracy would be better for chords of more spectrally dense harmonic complex stimuli than chords of pure tones. A trend for a main effect of stimulus type (pure tones, odd harmonic and full harmonic complexes) was reported for pitch matching accuracy in Section 2.4. To explore this further, separate within group ANOVAs for each musician group showed a significant difference in pitch matching accuracy between stimulus types for the AP musician group only, *F*(2,16) = 6.27, *p* < 0.01. Repeated planned contrasts revealed that pitch matching accuracy by AP musicians for chords of odd harmonic complexes (mean = 76.2, SD = 22.4) was significantly higher than for chords of pure tones (mean = 70.5, SD = 24.0), and although pitch matching accuracy for chords of full harmonic complexes was better than chords of odd harmonic complexes (mean = 77.8, SD = 20.0), this difference was not significant. These results are consistent with AP musicians possessing chord templates with more finally tuned spectral information than QAP and RP musicians, who showed no differences in accuracy across stimulus type.

Figure 3 shows that the pitch matching accuracy by AP musicians was greater than 50% for all target pitches of the 3-pitch chords except for the middle pitch of the very uncommon flattened and augmented fifth triads (4 and 6, and 4 and 8 chords). Figure 4 also shows histograms for these chords contrasted with the most familiar chord, the major triad (4 and 7). The histograms reveal systematic pitch matching errors by AP musicians for the middle pitch of the flattened and augmented fifth triads that are likely due to inversion errors caused by ambiguity in the position of the root pitch of these chords. In other words, the AP musicians appear to have systematically misclassified these chords, and so symbolically deduced an incorrect middle pitch on a significant number of trials. It is likely that AP musicians rely more heavily on their internal target pitch representation than subsequent presentations of the target stimulus, leading to more distinct error distributions than observed for QAP and RP musicians.

## 4. Discussion

This study shows a systematic effect of target stimulus context on the pitch matching performance of AP musicians. The dependency of AP and QAP pitch matching accuracy on chord familiarity and the location of the target pitch in the chord suggest that AP and QAP musicians first encode the identity of the chord in association with its highest pitch, rather than independently labeling each pitch in the chord before deducing the chord identity.

McLachlan and colleagues [4,5] proposed that RP musicians employ spectral recognition mechanisms to identify chords and the approximate pitch of their highest components prior to refinement of pitch representations by periodicity information. The current data suggest that AP musicians share this initial processing step with QAP and RP musicians. This is consistent with enhanced pitch naming performance by QAP musicians for more familiar instrumental timbres [40], and the finding that AP and RP musicians possess similar ability to recall stimulus timbre [47].

The finding that AP musicians were better able to resolve 2-semitone chords than QAP and RP musicians is consistent with the proposition that AP ability requires sufficient spectral resolution to discriminate semitone differences in tuning in order to separately identify and verbally label each pitch in the western chromatic scale. The pitch resolution of RP musicians for 2-pitch stimuli was found to be close to a critical bandwidth, which is slightly greater than two semitones at the frequency range of the stimuli used in this study. Spectral resolution is enhanced at various stages in the auditory pathways by lateral inhibition mechanisms, which could adapt to enable AP musicians to achieve semitone resolution with training.

### Absolute Pitch in the Dual Mechanism Model of Pitch

**Figure 5:**
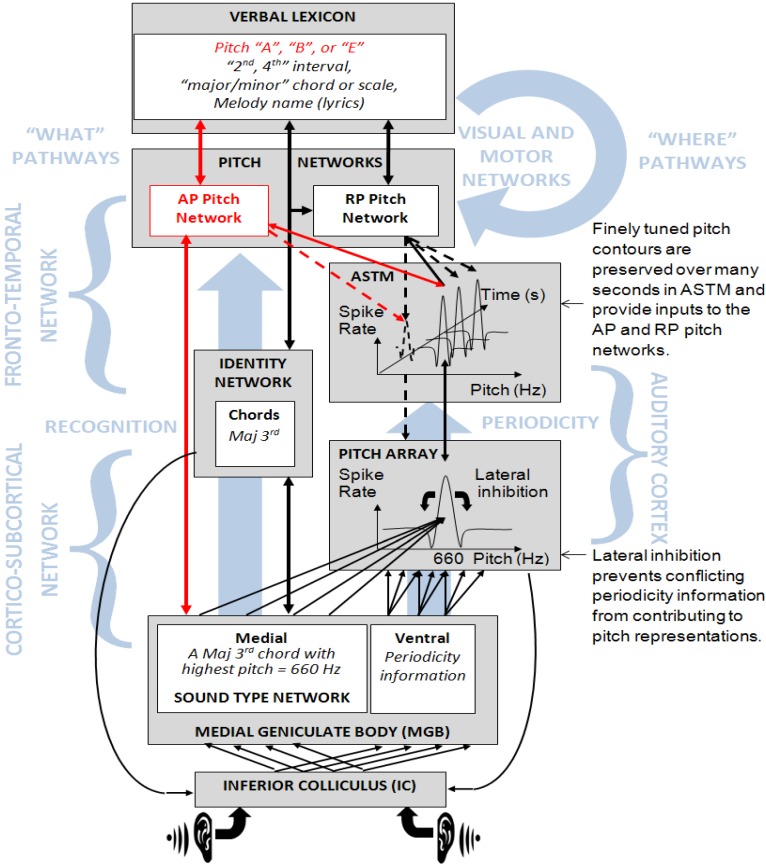
The dual mechanism model of AP in the context of concurrent pitch processing. Recognition mechanisms that involve the sound type and identity networks including verbal labeling are shown on the left of the figure. Periodicity processing through the auditory core is shown on the right. Spectral processing in the Inferior Colliculus (IC) provides inputs to the chord identity, RP (*black*) and AP (*red*) pitch networks, and the pitch array. Spectral recognition mechanisms prime one pitch of the chord in the pitch array, and chord identities prime pitch expectancies for the other pitches in auditory short-term memory (ASTM) (*dashed arrows*) via the RP pitch network. ASTM can also be directly primed by spectral recognition mechanisms in AP musicians.

Building on these findings and the two-component model of AP [33], we now present a more detailed neurocognitive account of AP based on a dual mechanism model of concurrent pitch processing [4] that has been expanded to include AP processing pathways. The dual mechanism model (Figure 5) is derived from “What” and “Where” pathway models of auditory processing [3,48] with the application of a two-component model of recognition [3,49] to AP. In Figure 5, the recognition (or “What” pathway on the left) leads to pitch labeling and priming of pitch representations in auditory short-term memory (ASTM), and periodicity processing (in the “Where” pathway on the right) refines pitch representations in the pitch array near the auditory core [50]. AP pitch templates may form by interaction of the identity network with refined pitch information in ASTM to facilitate fine grained pitch labeling templates. The model also allows for stimulus frequency information at the resolution of spectral encoding on the auditory nerve to be associated with sound identities that regularly occur in a listener’s environment at a fixed frequency, even in the absence of AP ability. For example, Smith and Schmuckler [51] reported that many people can recognize the correct pitch of phone dial tones at the frequency resolution of the auditory nerve (>70% correct for intervals within about three semitones of 350 and 440 Hz stimuli). Similarly the model can explain the ability of people to sing a popular melody that is predominantly heard in its original recorded version at close to the correct key [52,53]. In contrast, AP musicians possess much finer pitch categories than are possible from spectral encoding on the auditory nerve alone because their pitch labels are also associated with higher resolution pitch representations that are generated by further periodicity processing in the “Where” pathway [47].

In the dual mechanism pitch model (Figure 5), it is proposed that lateral inhibition within the pitch array in the auditory cortex prevents periodicity information at sub-harmonic frequencies of component tones from influencing pitch representations [2]. This has the consequence that only one pitch representation can form in the auditory core at any one time, and the listener must shift attention across component pitches in a chord during its presentation to generate a detailed representation of the chord in ASTM. Given the western music tradition of placing melodies at the highest pitch of chord accompaniment this likely explains the better pitch matching performance for the highest component pitch of chords across all three musician groups.

Most musicians develop the ability to make RP judgments, and generate accurate melodic expectancies based on long-term memory templates for common pitch intervals, melodies, and scales that form in relation to pitch representations in ASTM [3,54]. In Figure 5, ASTM is depicted as a store of higher level auditory representations (such as melodic contours) that can maintain multiple pitch traces concurrently [4]. In ASTM the refined pitch estimations generated in the auditory core from periodicity information (the “Where” pathway in Figure 5) are bound with a source identity that was computed in the parallel “What” pathway on the left of Figure 5. Pitch interval templates can be verbally labeled, and may form strong associations with chord templates (derived from spectral information) that share the same verbal label. For instance, a minor 3rd chord (concurrent pitch presentation) has the same label as a minor 3rd pitch interval (sequential pitch presentation). These RP templates could allow musicians to generate accurate expectancies for concurrent pitches if they have recognized the chord, and have an accurate pitch height representation of one of its pitches (typically the highest pitch) [4,5]. During stimulus presentation, musicians may choose to attend to expectations for the lower pitches that are generated by application of RP templates, thereby priming the pitch array to integrate periodicity information associated with a lower frequency pitch (Figure 5). This process may be facilitated by sub-vocalization to assist the maintenance of pitch representations in ASTM.

The model in Figure 5 proposes that AP pitch labels may be rapidly activated by recognition mechanisms that form the initial pitch estimate from spectral information, and then prime ASTM at the associated western musical pitch. In AP musicians the note label has also been associated with periodicity information in previous exposures, so finer pitch representations are rapidly generated in ASTM than could be achieved by spectral processing alone. This explanation requires that recognition mechanisms can estimate pitch within the resolution of one semitone to enable rapid activation of labels for pitch chroma. In support of this, Moore [16] reported pitch discrimination limens within one semitone for single pitch stimuli of just 12.5 ms duration (that could contain very little periodicity information).

Evidence for more rapid, spectral based pitch processing in AP musicians has also been reported in neurophysiological studies. P300 auditory evoked EEG responses were found to have smaller amplitudes and shorter latencies in AP musicians than non-AP musicians [55,56]. The planum temporale, a region associated with ASTM and P300 EEG responses [3], has a smaller volume in the right hemisphere compared to the left in AP musicians [40,57,58]. Since fine pitch processing generally occurs in the right hemisphere [59], a reduced short term memory load associated with fine pitch processing of melodies by AP musicians is consistent with AP musicians requiring fewer neural resources in the right planum temporale compared to non-AP musicians. In other words, just as object identification mechanisms have been shown to support working memory tasks [60], verbal associations in AP may support pitch representations in ASTM. Given linguistic processing generally occurs in the left hemisphere, this is also consistent with activation of the left posterior superior temporal gyrus during AP pitch naming tasks [40,57].

The proposition that AP musicians could accurately prime pitch representations from spectral information using AP templates is also consistent with the observation that AP musicians make slower and less accurate pitch interval identifications for non-standard pitches such as quartertones [61,62]. This may reflect inaccurate priming of non-standard pitches in ASTM by AP pitch templates that are centered on standard musical pitches in the Western chromatic scale, but nevertheless are rapidly activated by non-standard pitches given the low resolution of spectral pitch cues.

RP musicians are able to accurately identify pitch intervals despite many seconds of intervening time between presentations of the component pitches [54]. Miyazaki [62] suggested that the development of RP networks suppresses the ability of RP musicians to apply a verbal label to an isolated fine pitch representation in ASTM (AP ability). So verbal labeling of pitch traces in ASTM by AP musicians may involve similar brain mechanisms and pathways as pitch interval identification in RP musicians, except that concrete, absolute pitch judgments are maintained in AP in favor of relative pitch judgments [63,64,65]. This is consistent with the observation of proximal fMRI activation in language processing regions in the left posterior superior temporal gyrus during AP pitch naming tasks and RP tonal classification tasks [40].

The emergence and maintenance of AP skill then likely reflects long-term organization of auditory cortex for the formation of pitch label templates via mechanisms of associative learning [3,37]. The onset of training that uses consistent tone-label mappings during a sensitive period early in life may provide the developmental context that promotes organization of the auditory cortex to support AP in individuals with this genetic disposition [41]. Once present, AP templates may then need to be maintained by ongoing exposure to consistent tone-label mappings through regular practice on a fixed pitch instrument [41]. The dual mechanism pitch model proposes that AP musicians refine and maintain pitch labeling templates from fine pitch representations available in ASTM. This does not preclude the use of RP information by AP musicians [66,67], nor does it preclude the development of AP skill independent of musical training, although the absence of strong social or environmental motivations to accurately label non-musical pitches would make the occurrence of non-musical AP very rare [47,68]. Rather, AP may support the processing of musical information that is consistent with learnt pitch categories [67], and interact with RP information in ASTM [66]. Furthermore, the dual mechanism pitch model is consistent with findings that suggest that motor mechanisms can also prime spatial pitch representations in ASTM [69].

In AP, verbal labels form nominal categories of individual pitch representations that may be associated with both spectral templates for pitches, instrument timbres and chords, and fine pitch representations in ASTM. This mechanism would allow spectrally encoded information to bypass the integration of periodicity information in the auditory core, leading to more rapid fine pitch processing by AP musicians [42], but only for pitches tuned to the notes of the western scale [61,62]. This could improve AP pitch matching performance for chords when attention may need to shift between constituent pitches, and allow AP musicians to maintain pitch representations for durations beyond the length of pitch traces in ASTM [68]. However, in this study it may have also led to pitch matching errors due to incorrect initial classifications of the stimuli that were not corrected because the AP musicians focused their attention on internally generated representations of the target pitches, rather than on subsequent presentations of the stimuli.

## 5. Conclusions

The finding that chord familiarity affects the ability of AP musicians to match concurrent pitches is consistent with the dual mechanism model of pitch processing in which the chord itself must be recognized prior to the processing of individual component pitches. This points to various pathways, by which sound recognition mechanisms may interact with pitch processing. The finding that pitch matching accuracy is better for the highest pitch of chords across all musician groups suggests that sub-cortical spectral recognition mechanisms may consistently prime the integration of periodicity information at the highest pitch of chords. Recognition mechanisms may also subserve cortical associative networks such as the verbal labeling of long-term memory templates that form in relation to fine pitch representations in ASTM. The finding that AP musicians were more accurate than other musicians at pitch matching in spectrally dense 2-semitone (major 2nd) chords is consistent with AP musicians possessing finer resolution spectral templates. Finer resolution spectral templates may explain how AP musicians can make accurate pitch judgments faster than RP musicians, who must rely on slower periodicity-based processing mechanisms.
